# Composite lymphoma composed of follicular lymphoma and nodal T-follicular helper cell lymphoma: report of 3 cases highlighting histopathologic zonation of each neoplastic component

**DOI:** 10.1093/ajcp/aqag012

**Published:** 2026-03-05

**Authors:** Yue Zhao, Ibrahim Hajjali, Yaping Ju, Luis Carrillo, Mark Feng, Qianze Dong, Dongjiang Chen, Imran Siddiqi, Endi Wang

**Affiliations:** Department of Pathology, First Hospital and College of Basic Medical Sciences, China Medical University, Shenyang, Liaoning, P. R. of China; Department of Pathology, Keck School of Medicine, USC, Los Angeles, CA, United States; Department of Pathology, Keck School of Medicine, USC, Los Angeles, CA, United States; Depatment of Pathology and Laboratory Medicine, Duke University Medical Center, Durham, NC, United States; Department of Pathology, Keck School of Medicine, USC, Los Angeles, CA, United States; Department of Pathology, Case Western Reserve University School of Medicine, Cleveland, OH, United States; Department of Pathology, Keck School of Medicine, USC, Los Angeles, CA, United States; Department of Pathology, Keck School of Medicine, USC, Los Angeles, CA, United States; Department of Pathology, Keck School of Medicine, USC, Los Angeles, CA, United States

**Keywords:** composite lymphoma, follicular lymphoma, nodal T-follicular helper cell lymphoma

## Abstract

**Objectives:**

Composite lymphoma (CL), composed of follicular lymphoma (FL) and nodal T-follicular helper cell lymphoma (nTFHL), is an uncommon and diagnostically challenging entity. We present a small series of such cases to characterize their clinicopathologic and diagnostic features.

**Methods:**

We retrospectively analyzed 3 CL cases compared with 6 control cases of FL with expanded reactive T-follicular helper cells.

**Results:**

Histologically, all 3 CL cases demonstrated geographic zonation of the 2 neoplastic components, with the B-cell lymphoma residing in follicle centers (B-zones) and the T-cell neoplasm confined to perifollicular/interfollicular areas (T-zones), in contrast to a predominantly (83%) intrafollicular distribution of T-follicular helper cells in the control cases. In all CL cases, FL was suggested by histopathologic features, and the diagnosis was supported by flow cytometry. All 3 cases (100%) showed cytologic atypia and immunophenotypic aberrancy in the T-cell component, whereas none (0%) were observed in the control group. In 2 cases (66.7%), scattered Epstein-Barr virus–positive cells were seen in the T-zone, suggesting latent infection in bystander cells, again compared to none (0%) in the control. Genomic sequencing was performed in 2 cases, both (100%) showing pathogenic mutations associated with nTFHL, while none (0%) of the controls showed such mutations. Biclonality was confirmed by B-cell and T-cell receptor gene rearrangement analyses in all 3 CL cases. All patients with CL presented with an aggressive clinical course.

**Conclusions:**

This series highlights the unique histopathologic characteristics of CL and underscores the importance of a multifaceted approach to diagnosis.

KEY POINTS

- Composite follicular lymphoma (FL) and nodal T-follicular helper cell lymphoma (nTFHL) is an extremely rare entity with significant diagnostic challenges.
- These composite lymphomas demonstrate distinct histologic zonation, with FL localized to follicle centers and nTFHL confined to the paracortex.
- Accurate diagnosis requires a comprehensive, multimodal approach incorporating morphology, flow cytometry, cytogenetics, and molecular analysis.

## INTRODUCTION

Two distinct lymphomas can sometimes occur in a single individual. When 2 or more lymphomas of different clonal origins are present in the same lymph node or extranodal site, the diagnosis of composite lymphoma (CL) is applied.^1^ The composition of CL varies, including combinations of Hodgkin lymphoma and non-Hodgkin lymphoma, different B-cell lymphomas, or different T-cell lymphomas. Composite lymphoma of B-cell and T-cell origins is infrequently encountered in diagnostic practice, and only rare cases have been described in the literature, either as case reports^1‐12^ or as small case series.^3^^,^^13^^,^^14^ In this latter category, previously reported cases have primarily included diffuse large B-cell lymphoma (DLBCL) occurring as a composite with either angioimmunoblastic T-cell lymphoma^5^^,^^6^^,^^15^ or peripheral T-cell lymphoma, not otherwise specified (PTCL-NOS).^13^^,^^16^ Additionally, rare cases of T-cell lymphoma have been reported to occur as a composite with other B-cell lymphoma, such as small lymphocytic lymphoma/chronic lymphocytic leukemia (SLL/CLL),^3^^,^^13^^,^^17^ marginal zone lymphoma,^13^^,^^18^ mantle cell lymphoma,^13^^,^^19^ or Hodgkin lymphoma.^20^ Conversely, follicular lymphoma (FL), a B-cell neoplasm, has been noted to form composites with other B-cell lymphomas, including classic Hodgkin lymphoma,^21^ mantle cell lymphoma,^22‐24^ SLL/CLL,^25^ marginal zone lymphoma,^26^ DLBCL,^27^ and nodular lymphocyte-predominant Hodgkin lymphoma.^28^ However, FL concomitant with a T-cell lymphoma is uncommon, with the literature limited to sporadic case reports.^29^^,^^30^ A CL composed of FL and nodal T-follicular helper cell lymphoma (nTFHL) is extremely rare, with only 1 case reported in the English literature,^4^ to the best of our knowledge. Due to its rarity, the histopathologic features of CL composed of B-cell and T-cell origin, particularly those involving FL and nTFHL, remain poorly characterized, and the diagnostic criteria have yet to be clearly defined. Herein, we present a retrospective analysis of 3 cases of CL composed of FL and nTFHL occurring in the same lymph nodes. Our aim is to summarize the key histopathologic features and highlight important considerations in diagnosing these complex cases.

## PATIENTS AND METHODS

### Case selection

This study was approved by the institutional review boards of the University of Southern California (USC) Keck Medicine/Norris Hospital and Duke University Medical Center. All 3 cases of CL in individual patients were reported within the past decade, including 2 cases (cases 1 and 2) from Duke University Medical Center and 1 case (case 3) from Keck Medicine of USC. All cases were retrospectively reviewed by authors E.W., L.C., and I.S., and diagnoses were confirmed according to the fifth edition of the World Health Organization Classification of Tumors of Hematopoietic and Lymphoid Tissues^31^ and the International Consensus Classification of Mature Lymphoid Neoplasms: A Report from the Clinical Advisory Committee.^32^ Clinical history and laboratory data, including radiologic imaging, serum lactate dehydrogenase, complete blood cell count, flow cytometric analysis, cytogenetic studies, molecular tests, and so forth, were collected and analyzed retrospectively. Cases of composite B-cell and T-cell lymphoma other than the combination of FL and nTFHL were excluded from this study.

## RESULTS

The clinical presentation, histopathologic features, immunophenotypic findings, cytogenetic and molecular data, and treatment and follow-up for 3 cases of CL composed of FL and nTFHL are summarized in Table 1. Correspondingly, data for 6 cases of FL with expanded reactive T-follicular helper cells are summarized in Table S1.

**Table 1:** Clinicopathologic Features of Composite Lymphoma Composed of Follicular Lymphoma and Nodal T-Follicular Helper Cell Lymphoma

Characteristic	Case 1^a^	Case 2	Case 3
**Age, y**	75	49	84
**Sex**	Male	Male	Female
**Clinical presentation**	Mild fatigue with neck swelling; no fever or weight loss	Chest pain and generalized lymphadenopathy	Generalized weakness for 2 months, then fever and abdominal pain
**Radiologic findings**	Generalized lymphadenopathy	Generalized lymphadenopathy and pleural effusion	Generalized lymphadenopathy and splenomegaly
**CBC (WBC/Hb/Plt)**	NA	8.5/11.8/205	20.6/9.2/46; neutrophils 87.5%
**Lymphocytosis**	NA	No	No (5.4%)
**Serum LDH, U/L**	NA	223	NA
**Biopsied nodal site**	Left neck (excision)	Right groin (excision)	Left axilla (core needle)
**Histopathology**	Zonation of 2 components	Zonation of 2 components	Zonation of 2 components
**B-zone**			
** B-cell distribution**	Follicular	Follicular	Follicular
** Histologic grade**	1	2	3B
** Proliferation index**	∼5%	∼60%	∼95%
** B-cell phenotype (IHC)**	CD20+, CD19+, CD79a+, PAX5+, OCT2+, CD10+, BCL6+, BCL2++, CD5–	CD20+, CD10+, BCL6+, BCL2–, BCL1–, CD5–	CD20+, PAX5+, BCL6+, CD10–, BCL2–, BCL1–, MUM1–, CD5–
**T-zone**			
** T-cell distribution**	Interfollicular	Perifollicular/interfollicular	Interfollicular
** Cytologic atypia**	Prominent with enlarged cells	Mild to moderate with small to intermediate cells with clear cytoplasm	Prominent with enlarged cells
** Microenvironment**	Angioimmunoblastic with eosinophilia	Angioimmunoblastic with eosinophilia	Angioimmunoblastic with eosinophilia
** Proliferation index**	∼35%	∼30%	65%
** T-cell phenotype (IHC)**	s/cCD3+, CD5+, CD20+, CD4+, BCL6+, PD1+, CD10+/–, CD8–, CD7–	cCD3+, CD5+, CD7+, CD4+, CD10+, BCL6+, PD1+, CD2–, CD8–	cCD3+, CD2+, CD5+, CD4+, BCL6+, PD1+, CD10+/–, ICOS+, CXCL13+, MUM1+, CD30+, CD7–, CD8–
** EBER CISH**	Positive in scattered interfollicular cells	Negative	Positive in scattered interfollicular cells
**Flow cytometry**	Both components detected	Both components detected	Both components detected
**Abnormal B cells (quantity/phenotype)**	37% CD20+, CD10+, CD19–, sIg–, CD5–, CD23–; small in size	17% CD20+, CD19+, sCD22+, sIg kappa+, CD10+, CD5–, CD23–, sIg λ–; small to medium in size	19% CD20+, CD19+, CD10–, CD5–, sIg–; large in size
**Abnormal T cells (quantity/phenotype)**	41% sCD3+, CD2+, CD5+, CD4+, CD8–, CD7–	9% CD2+, CD5+, CD7+, CD4+, CD10+, sCD3–, CD8–	18% cCD3+, CD2+, CD5+, CD4+, CD279 (PD1)+, CD10+/–, sCD3–, CD7–, CD8–, TRBC1–
**B-cell clonality**	Clonal *IGH*	Clonal *IGK*	Clonal *IGH, IGK*
**T-cell clonality**	Clonal *TCRG*	Clonal *TCRG*	Clonal *TCRG, TCRB*
**FISH analysis**	*IGH::BCL2* (78%) in B-zone; gains of *IGH* or tetrasomy 14 (54%) in T-zone	NA	Negative for rearrangements of *BCL2*, *BCL6*, and *MYC*
**NGS analysis**	NA	*TET2* E1376fs (7%); *RHOA* G17V (9%)	*DNMT3A* W581^a^ (26.1%); *TET2* Q1020^a^ (24%)
**Bone marrow biopsy**	No lymphomatous involvement	No lymphomatous involvement	Involved by large B-cell lymphoma
**Treatment**	Etoposide + CHOP	Etoposide + CHOP; then romidepsin, azacytidine, and decitabine after AITL relapse	Palliative care
**Follow-up, mo**	6	24	3
**Outcome**	Progressive lymphadenopathy; then loss of follow-up	Progressive lymphadenopathy; then loss of follow-up	Loss of follow-up

Abbreviations: –, negative; +, positive; +/–, partially positive; AITL, angioimmunoblastic T-cell lymphoma (nodal T-follicular helper cell lymphoma, angioimmunoblastic type); CBC, complete blood cell count; cCD3, cytoplasmic CD3; CHOP, cyclophosphamide, doxorubicin (adriamycin), vincristine (oncovin), and prednisone; EBER CISH, Epstein-Barr virus-encoded RNA chromogenic in situ hybridization; FISH, fluorescence in situ hybridization; Hb, hemoglobin (g/L); *IGH*, immunoglobulin heavy chain gene; *IGK*, immunoglobulin kappa light chain gene; IHC, immunohistochemistry; LDH, lactate dehydrogenase; NA, not available; NGS, next-generation sequencing analysis; Plt, platelets (× 10^9^/L); sCD3, surface CD3; sIg, surface immunoglobulin light chain; *TCRB*, T-cell receptor beta gene; *TCRG*, T-cell receptor gamma gene; TRBC1, T-cell receptor beta chain constant region 1; WBC, white blood cells (× 10^9^/L).

a This case has been previously reported (Tanaka et al.).

### Clinical presentation

Among the 3 patients with CL, 2 were men and 1 was a woman. The median age at diagnosis was 75 years, ranging from 49 to 84 years. All 3 patients had B-symptoms at initial presentation. One patient (case 1) reported chronic fatigue, while the other 2 patients (cases 2 and 3) experienced malaise, body pain, and fever. Generalized lymphadenopathy was identified in all patients by computed tomography (CT) scan. The complete blood cell count data were available for 2 cases (cases 2 and 3), both showing mild or moderate anemia. Case 3 also exhibited moderate thrombocytopenia and marked left-shifted neutrophilia, which may have resulted from bone marrow involvement by lymphoma or a cytokine-mediated effect. None of the patients demonstrated peripheral lymphocytosis or circulating lymphoma cells. Of note, patient 2 underwent fine-needle aspiration and core needle biopsy of the right cervical lymph nodes. Flow cytometric analysis performed on these samples revealed a small monoclonal population of CD10-positive B cells. Although these findings were suspicious for a clonal B-cell lymphoproliferative disorder in a T-cell–rich background, neither biopsy specimen was sufficient for a definitive diagnosis. This patient subsequently developed progressive mediastinal and cervical lymphadenopathy, with radiologic evidence of airway compression and clinical presentation of stridor. Further CT imaging showed progression of lymphadenopathy involving the abdomen, retroperitoneum, pelvis, external iliac chain, and groin, prompting excision of a right groin lymph node. Among the 3 cases, the final diagnosis of CL was made via excisional biopsy in 2 patients (cases 1 and 2) and via core needle biopsy in the third. Staging bone marrow biopsy showed B-cell lymphoma involvement in 1 patient (case 3), with no evidence of bone marrow disease in the other 2 patients. Notably, patient 2 initially responded to therapy with resolution of symptoms and lymphadenopathy but relapsed approximately 1 year later with recurrent B-symptoms. Computed tomography imaging revealed progressive lymphadenopathy throughout the neck and superior mediastinum. Subsequent excisional biopsy of a left supraclavicular lymph node demonstrated nTFHL without flow cytometric or histopathologic evidence of FL. Restaging positron emission tomography (PET)/CT showed generalized lymphadenopathy involving the tonsils, cervical region, thorax, mediastinum, and retroperitoneum, with a standardized uptake value ranging from 3 to 6.5.

### Histologic evaluation and immunohistochemical analysis

**Figure 1:**
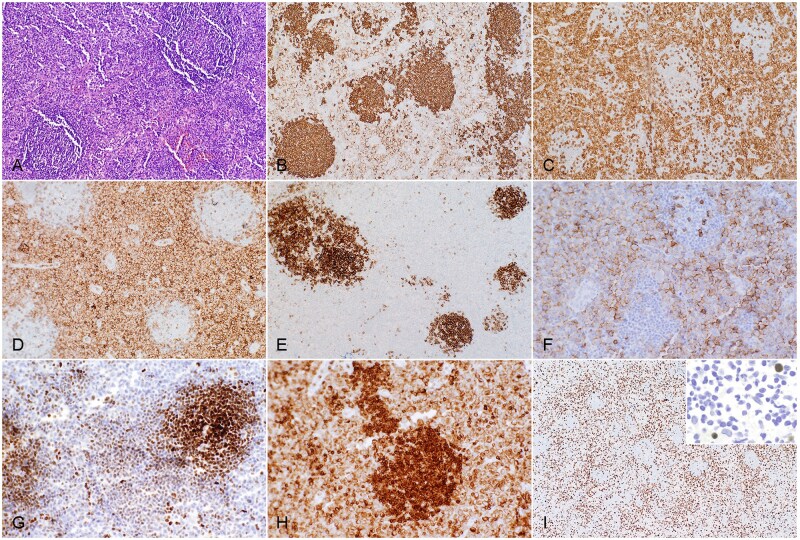
Histopathologic evaluation of composite follicular lymphoma and nodal T-follicular helper cell lymphoma in axillary lymph node biopsy (case 1). **A**, Section of the biopsy specimen exhibits vague lymphoid follicles and expansion of interfollicular areas. Hematoxylin and eosin stain (×100). **B**, CD20 stain (×100). **C**, CD3 stain (×100). **D**, CD4 stain (×100). **E**, CD10 stain (×100). **F**, PD1 stain (×200). **G**, BCL6 stain highlights germinal center cells with stronger staining and many interfollicular cells (abnormal T cells) (×200). **H**, BCL2 stain is positive in both follicular lymphocytes and interfollicular T cells with apparent overexpression in follicular B cells (×200). **I**, Ki-67 stain highlights an increased proliferation index in an interfollicular pattern (×40). Inset shows rare cells with Epstein-Barr virus latent infection. Epstein-Barr virus-encoded RNA chromogenic in situ hybridization (×400).

All 3 cases of CL showed follicular growth pattern of B-cell proliferation, with histologic grade 1 (case 1), grade 2 (case 2), and grade 3B (case 3), respectively. None of the cases displayed cellular polarity. In case 1, histologic examination of the left cervical lymph node showed scattered lymphoid follicles with expanded interfollicular areas (Figure 1A). The follicles consisted of monomorphic small mature lymphocytes with centrocyte-like nuclear features, without cellular polarity or tingible body macrophages. Mantle zones were either markedly attenuated or essentially absent. The interfollicular (T-zone) cells were intermediate to large in size, with round to oval nuclear contours, vesicular chromatin, and scant to moderate cytoplasm. They were admixed with small lymphocytes, plasma cells, and eosinophils in a background rich in endothelial venules. Immunohistochemical analysis demonstrated findings as follows: CD20 highlighted lymphoid follicles with strong staining and was also positive, albeit weaker, in interfollicular cells (Figure 1B). The T-cell lineage of interfollicular cells was confirmed by their expression of CD3 (Figure 1C), CD5, CD7 (partial), and CD4 (Figure 1D). CD8 stained only scattered interfollicular lymphocytes. CD10 was strongly positive in follicular B cells and also marked scattered interfollicular lymphocytes (Figure 1E). In addition, interfollicular cells were positive for PD1 (Figure 1F) and BCL6 (Figure 1G). BCL2 showed dual staining intensities, with follicular B cells displaying stronger staining than interfollicular T cells (Figure 1H), suggesting its overexpression in follicular B cells. Follicular B cells were also positive for CD19, CD79a, PAX5, and OCT2, while interfollicular lymphocytes were negative for these markers, confirming their T-cell lineage despite aberrant CD20 expression. Ki-67 demonstrated an elevated proliferation index in the interfollicular areas, while follicular nodules showed a lower index (Figure 1I). Epstein-Barr virus (EBV) small RNA in situ hybridization (EBER CISH) revealed scattered positive cells in interfollicular areas, suggestive of latent EBV infection in bystander cells (Figure 1I, inset).

**Figure 2:**
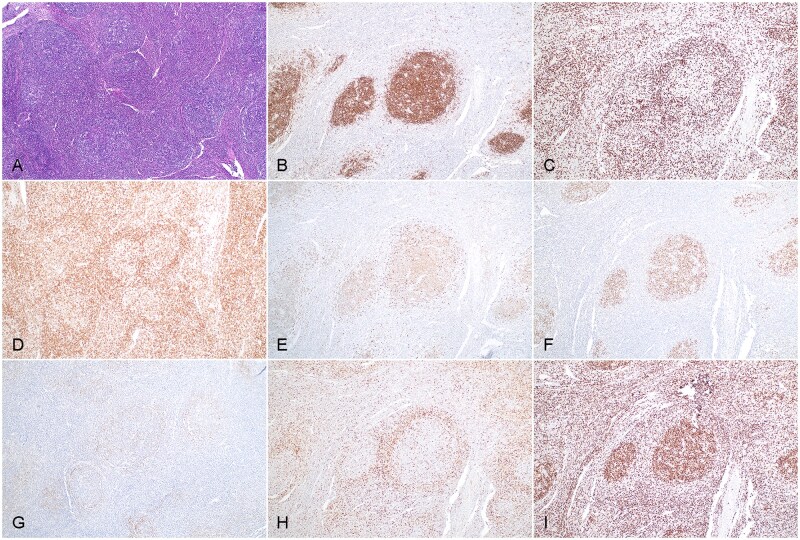
Histopathologic evaluation of composite follicular lymphoma and nodal T-follicular helper cell lymphoma in an inguinal lymph node biopsy specimen (case 2). **A**, Section of the biopsy specimen shows increased lymphoid follicles with poorly defined germinal centers. Hematoxylin and eosin stain (×100). **B**, CD20 stain (×100). **C**, CD3 stain (×100). **D**, These T cells are largely restricted to the CD4 subset. CD4 stain (×100). **E**, CD10 stain shows positivity in follicular lymphocytes and in perifollicular cells with stronger staining (×100). **F**, BCL6 stain highlights germinal center cells and scattered perifollicular cells (T cells) (×100). **G**, PD1 stain highlights perifollicular T cells with weak staining (×100). **H**, BCL2 stain is positive in perifollicular T cells, but follicular B cells appear to have negative or weak staining. Note the poorly defined follicle centers (×100). **I**, Ki-67 stain demonstrates a high proliferation index in follicle centers without cellular polarity (×100).

**Figure 3:**
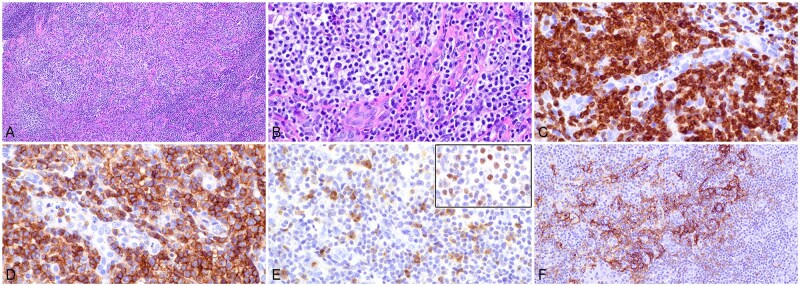
Histopathologic evaluation of relapsed nodal T-follicular helper cell lymphoma in a left supraclavicular lymph node (case 2). **A**, A low magnification of a hematoxylin and eosin stain (×100). **B**, A high magnification. Hematoxylin and eosin stain (×400). **C**, CD3 stain highlights atypical lymphocytes. Note the negative vascular endothelial cells (×400). **D**, These T cells are largely restricted to the CD4 subset. CD4 stain (×400). **E**, CD10 stain shows a scattered increase in positive cells (×400). Inset, BCL6 stain showing its expression in atypical cells (×400). **F**, CD23 stain highlights extrafollicular extension of the follicular dendritic meshwork and entrapped atypical T cells (×200).

Case 2 showed similar histologic features to case 1, except that the T-follicular helper cells had a predominantly perifollicular distribution and less conspicuous cytologic atypia. The excised right inguinal lymph node section showed an effaced nodal architecture with vague nodular/follicular proliferation (Figure 2A). Follicles had poorly defined centers with an attenuated mantle zone. Cellular polarity was absent, but rare tingible body macrophages were present. Interfollicular expansion was focal. Immunohistochemical analysis showed the findings as follows: CD20 highlighted follicle centers (Figure 2B), and CD3 was positive in perifollicular and interfollicular lymphocytes (Figure 2C). Perifollicular T cells coexpressed CD4 (Figure 2D), CD10 (Figure 2E), BCL6 (Figure 2F), and PD1 (Figure 2G), while B cells within follicle centers were positive for CD10 (Figure 2E) and BCL6 (Figure 2F) but negative for BCL2 (Figure 2H). The proliferation index was estimated at 60% in follicle centers and at 30% in perifollicular/interfollicular (T-zone) areas (Figure 2I). CD21 and CD23 highlighted follicular dendritic meshworks expanding beyond follicular boundaries. EBER CISH was negative. At relapse (1 year later), the left supraclavicular lymph node showed completely effaced nodal architecture with diffuse atypical lymphocyte proliferation (Figure 3A). These lymphocytes were small to medium in size, with slightly irregular nuclear contours, condensed chromatin, and moderate amount of clear cytoplasm (Figure 3B). Multiple foci showed clusters of these abnormal lymphocytes, along with scattered eosinophils and prominent endothelial venules. Immunohistochemical analysis demonstrated the abnormal lymphocytes were positive for CD3 (cytoplasmic staining) (Figure 3C), CD4 (Figure 3D), partial CD10 (Figure 3E), BCL6 (Figure 3E, inset), and PD1; CD23 (Figure 3F) highlighted extrafollicular expansion of follicular dendritic meshwork associated with abnormal T cells.

**Figure 4:**
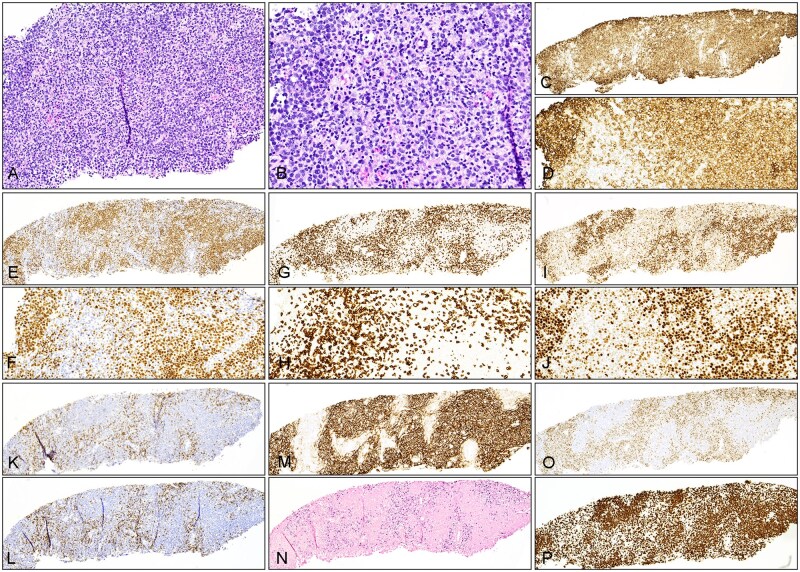
Histopathologic evaluation of composite follicular lymphoma and nodal T-follicular helper cell lymphoma in a left axillary lymph node biopsy specimen (case 3). **A**, Section of the biopsy specimen shows needle core with abnormal lymphoid proliferation with the dark zone (B-cell zone or follicle center) and pale zone (T-zone). Hematoxylin and eosin stain (×100). **B**, The lymphocytes in the dark zone (in the left and upper right corners) are monomorphic and medium to large in size, with a scattered increase in apoptosis or tingible body macrophages. Note atypical lymphoid cells in the pale zone with increased size and irregular nuclear contours, as well as scattered eosinophils. Hematoxylin and eosin stain (×200). **C**, CD20 stain highlights predominantly lymphoid follicles (×40). **D**, This higher magnification shows medium to large B cells within follicles. CD20 stain (×200). **E**, **F**, PAX5 stain highlights predominantly follicular growth of abnormal B cells and relatively attenuated interfollicular areas (**E**, ×40; **F**, ×100). **G**, **H**, CD3 stain demonstrates staining of interfollicular T cells highlighting negative (B-cell) nodules (**G**, ×40). Note the enlarged cellular contours of T cells (**H**, ×200). **I**, **J**, BCL6 stain shows positivity in follicular lymphocytes with uniform strong staining and in interfollicular cells with weak staining (**I**, ×40; **J**, ×200). **K**, PD1 stain highlights interfollicular cells (T cells) (×40). **L**, ICOS stain shows staining of interfollicular cells (×40). **M**, CD21 stain highlights follicular dendritic meshworks that appear expanded into the T-zone (×40). **N**, Epstein-Barr virus-encoded RNA chromogenic in situ hybridization demonstrates staining in interfollicular distribution (×40). **O**, BCL2 stain is positive in interfollicular cells, highlighting BCL2-negative lymphoid follicles (×40). **P**, Ki-67 stain shows increased proliferation index in interfollicular areas. Note the high proliferation index in follicle centers without cellular polarity (×40).

In case 3, the core needle biopsy specimen of the left axillary lymph node demonstrated vague nodular/follicular lymphocyte proliferation (Figure 4A). Follicles had poorly defined centers and lacked clear mantle zones. Although cellular polarity was absent, apoptotic activity appeared increased (Figure 4B). Interfollicular areas showed expansion with numerous atypical lymphocytes admixed with small lymphocytes, histiocytes, and eosinophils. Immunohistochemical analysis showed the findings as follows: CD20 (Figure 4C and D) and PAX5 (Figure 4E and F) highlighted follicular B cells, and CD3 (Figure 4G and H), CD2, and CD5 were positive in interfollicular lymphocytes, which were heterogeneous in size with increased medium- to large-sized cells. In addition, the interfollicular T cells coexpressed CD4, CD10 (partial staining), BCL6 (weak to moderate staining; Figure 4I and J), PD1 (Figure 4K), ICOS (Figure 4L), and CXCL13. CD21 highlighted expanded and distorted follicular dendritic meshworks (Figure 4M), and EBER CISH showed scattered positive cells in interfollicular areas (Figure 4N). While B cells in follicles were highlighted by BCL6 (Figure 4I and J), they were negative for BCL2 (Figure 4O). Ki-67 showed a proliferation index of ∼95% in follicles and 60% to 70% in interfollicular areas (T-zone) (Figure 4P).

### Flow cytometric analysis

**Figure 5:**
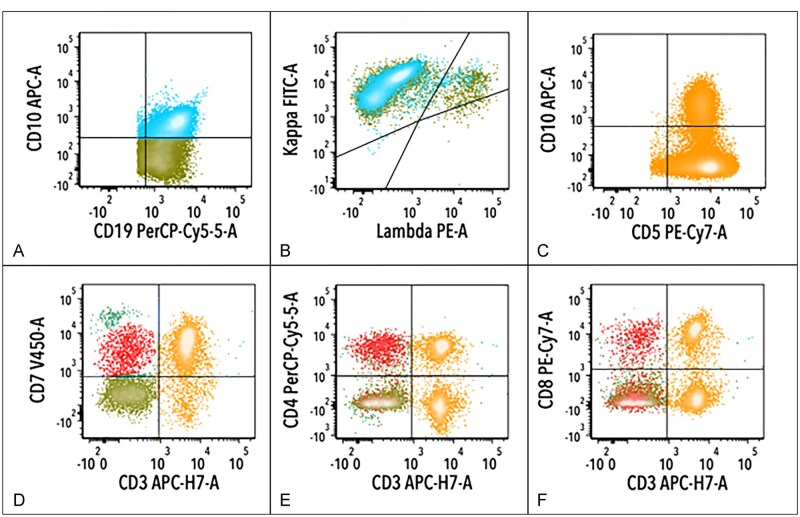
Flow cytometric analysis of follicular lymphoma and nodal T-follicular helper cell lymphoma in an inguinal lymph node biopsy specimen (case 2). **A**, **B**, The analysis, with the gate set on CD19-positive events, demonstrates a monoclonal B-cell population (blue colored events) with partial CD10 (**A**) and κ light chain restriction (**B**). **D-F**, The analysis, with the gate set on bright CD45-positive (lymphoid) events, demonstrates T cells with a subset population positive for CD10 (**C**), loss of surface CD3 (red events in **D**), expression of CD4 (**E**), and partial staining for CD8 (double positive T cells) (**F**).

All 3 cases of CL underwent flow cytometric analysis, and all demonstrated a phenotypically abnormal T-cell population, in addition to a monoclonal B-cell population (Figure 5A and B; case 2). In all 3 cases, phenotypic aberrancies were observed in the CD4-positive T-cell population (Figure 5C-F), including loss of CD7 in 2 (cases 1 and 3), loss of surface CD3 (Figure 5D-F) in 2 (cases 2 and 3), expression of CD10 (Figure 5C) in 2 (cases 2 and 3), and expression of CD20 in 1 (case 1) case(s).

### Cytogenetic studies

Fluorescence in situ hybridization (FISH) analysis was performed in 2 CL cases (cases 1 and 3). In case 1, *IGH::BCL2* fusion was detected in 78% of interphase nuclei in targeted areas of small cells (presumably neoplastic follicles) on paraffin-embedded sections. In the T-zone (areas of larger cells), FISH showed extra *IGH* signals suggestive of *IGH* amplification or trisomy/tetrasomy 14 in 54% of interphase nuclei. In case 3, FISH was performed on bone marrow involved by large B-cell lymphoma. No rearrangements of *BCL2*, *BCL6*, or *MYC* were detected. Chromosome analysis of the bone marrow demonstrated complex cytogenetic abnormalities in lymphoid-stimulated metaphase cells, although no evidence of t(14; 18) or translocation involving 3q27.3 (*BCL6* locus) was observed.

### Molecular diagnostic analysis

*IGH/K* rearrangement and T-cell receptor gene (*TCR*) rearrangement analyses were performed on paraffin-embedded tissue from all 3 CL cases. All demonstrated clonal rearrangement of both immunoglobulin and TCR genes, confirming B- and T-cell clonalities.

Next-generation sequencing (NGS) using a lymphoid-directed panel of gene targets was performed in 2 CL cases (cases 2 and 3). In case 2, NGS (performed on the relapsed nTFHL tissue) revealed pathogenic mutations in *TET2* (p.E1376fs) and *RHOA* (p.G17V), with variant allele frequencies (VAFs) of 7% and 9%, respectively. In case 3, pathogenic mutations were identified in *DNMT3A* (p.W581*) and *TET2* (p.Q1020*), with VAFs of 26.1% and 24%, respectively. The mutation profiles in both cases supported the diagnosis of nTFHL. Pathogenic mutations typically associated with FL were not present in either of the CL cases, although in case 2, it could be explained by the absence of detectable FL in this lymph node sample with relapsed nTFHL.

### Treatment and clinical course

Of the 3 patients with CL, 2 were treated with cyclophosphamide, doxorubicin, vincristine, etoposide, and prednisone (CHOEP), a regimen primarily targeting T-cell lymphomas. The third patient received palliative care due to poor clinical status and advanced age. Both patients who underwent chemotherapy exhibited treatment resistance and evidence of disease progression. In case 1, the patient had excessive fatigue, anorexia, and a weight loss of approximately 35 pounds. A subsequent PET/CT scan revealed persistent generalized lymphadenopathy, with multiple lymph nodes showing increased fluorodeoxyglucose (FDG) uptake. Hematopoietic stem cell transplantation was considered but not pursued due to the patient’s age, poor performance status, and comorbidities. He was referred to another medical institution as a candidate for a clinical trial but was eventually lost to follow-up. In case 2, the patient first received 1 cycle of rituximab, cyclophosphamide, and dexamethasone for cytoreduction, followed by 6 cycles of CHOEP. One month after chemotherapy, PET/CT demonstrated decreased generalized lymphadenopathy with no FDG activity above background, although multiple enlarged lymph nodes were still radiologically evident. While interim assessment did not show significant residual disease, the patient presented approximately 1 year later with fatigue and shortness of breath. Computed tomography of the neck and chest revealed progressive lymphadenopathy in the neck and superior mediastinum. An excisional biopsy specimen of the left supraclavicular lymph node confirmed nTFHL with no evidence of FL. Restaging PET/CT showed generalized lymphadenopathy involving the tonsils, cervical region, thorax, mediastinum, and retroperitoneum with standardized uptake value values ranging from 3 to 6.5. The patient was treated subsequently with 3 cycles of romidepsin, 5 cycles of azacytidine, and 2 cycles of decitabine. Although initial clinical response was noted, the patient relapsed again with nTFHL approximately 1 year after salvage chemotherapy. The treatment was readjusted accordingly, but the patient was ultimately lost to follow-up, with the last clinic visit recorded 24 months after diagnosis. The remaining patient, who received only palliative care, did not return for scheduled follow-up appointments and was also lost to follow-up (Table 1).

## DISCUSSION

Composite lymphoma is an uncommon lymphoproliferative disorder, comprising fewer than 1% of all lymphomas.^1^^,^^14^^,^^33^ The most common reported type of CL involves a combination of Hodgkin lymphoma and non-Hodgkin lymphoma.^33^ Composite lymphoma consisting of mature T-cell and mature B-cell lymphomas has also been described, most often involving DLBCL in combination with either angioimmunoblastic T-cell lymphoma (AITL)^5^^,^^6^^,^^15^ or PTCL-NOS.^13^^,^^16^ Prior to the fifth edition of the World Health Organization (WHO) Classification, FL was reported primarily as a component of composite B-cell lymphoma.^21‐28^ In contrast, FL in combination with a mature T-cell lymphoma is extremely rare, and relevant literature has only recently emerged.^29^^,^^30^ Yang et al.^9^ recently reported a case of FL coexisting with anaplastic large cell lymphoma, while Raychaudhuri et al.^0^ described EBV-positive nodal natural killer/T-cell lymphoma arising in a background of FL. In contrast, our current series demonstrates concurrent FL and nTFHL within the same lymph node biopsy specimen for each patient. Notably, the 2 neoplastic components displayed zonal compartmentalization: FL was confined to follicular areas (B-zone), while nTFHL was present in the interfollicular (cases 1 and 3) or perifollicular (case 2) region (T-zone). This T-zone distribution of nTFHL stands in striking contrast to the intrafollicular localization of reactive T-follicular helper cells observed in some cases of FL (supplemental data). Of the 3 cases, only case 1 showed clear histopathologic features supporting a diagnosis of T-cell lymphoma, including enlarged interfollicular T cells with a T-follicular helper cell (TFH) phenotype and aberrant CD20 expression, alongside concurrent FL with BCL2 overexpression. In cases 2 and 3, the diagnosis of nTFHL was made via a multifaceted approach involving histopathologic features of T-zone infiltration, flow cytometric evidence of abnormal T cells, clonal rearrangement of *TCR*, and NGS mutation profiles characteristic of nTFHL. In these 2 cases, follicular B cells lacked BCL2 overexpression, but the neoplastic nature of B-cell proliferation was supported by other histopathologic features, flow cytometric detection of monoclonal B cells, and clonal B-cell receptor gene rearrangement. In all 3 CL cases, the neoplastic T-cell component was confined to the T-zone without extensive expansion, likely a result of the containment effect exerted by the neoplastic B-cell component. This phenomenon has also been observed in cases of PTCL-NOS coexisting with FL^30^ or mantle cell lymphoma with a follicular pattern,^13^ suggesting a strong homing preference of neoplastic B cells for follicular niches. Importantly, the salient follicular presentation of the B-cell component with a contained T-zone may obscure recognition of the T-cell neoplasm, particularly in nTFHL, where cytologic atypia can be subtle. In our study, flow cytometry was instrumental, detecting aberrant T-cell populations in all 3 CL cases, including those lacking overt cytologic atypia (cases 2 and 3). Incidental detection of biclonal lymphoid populations prompted further scrutiny of histology and the addition of molecular testing, leading to the diagnosis of CL in cases 2 and 3.

Histologic diagnosis of nTFHL, particularly the angioimmunoblastic subtype, is well known to be difficult due to the heterogeneous morphology of neoplastic T cells and polymorphous infiltration of reactive elements. The presence of coexisting FL further complicates the diagnosis by restricting the T-cell component to the interfollicular compartment. In our experience, detection of a discrete population of the abnormal T-cell population (eg, aberrant CD20 expression in case 1 or pan–T-cell antigen loss in cases 1-3) either by immunohistochemistry or flow cytometry provides the most compelling evidence for a concurrent T-cell neoplasm, particularly in the context of a coexisting FL. Molecular analysis can also provide convincing evidence. Pathogenic mutations in *TET2*, *DNMT3A*, and *RHOA*, frequently found across different subtypes of nTFHL, have been observed in 73%, 36%, and 69% of the cases, respectively.^34^^,^^35^ Recurrent mutations in these genes are uncommon in other lymphomas,^36^^,^^37^ making them useful diagnostic markers. However, caution is required, as somatic mutations in *TET2* and *DNMT3A* may occur as early events in hematopoiesis. Variant allele frequencies should be interpreted in the context of histologic and immunophenotypic findings to assign the lineage origin of each mutation. Clonal *TCR* gene rearrangement may provide additional support for a T-cell neoplasm but carries a significant false-positive rate in some settings.^38^ Thus, it should only be considered supportive when other diagnostic features suggest T-cell lymphoma. Latent EBV infection of bystander B cells in the T-zone, as seen in cases 1 and 3, can also support a diagnosis of nTFHL, particularly in AITL,^39^ and was not observed in those cases of FL with expanded T-follicular helper cells (supplemental data). Additional distinguishing features included higher proliferation indices (>30%) and cytologic atypia among interfollicular T cells.

The pathogenesis of composite B-cell and T-cell lymphoma remains unclear.^13^ Are these truly separate neoplasms occurring coincidentally, or do they arise from a shared pathogenic mechanism? Some data suggest a predisposition for certain B-cell lymphomas, like DLBCL, to co-occur with T-cell neoplasms, particularly AITL, often in the context of EBV infection.^13^^,^^40^ In many such cases, EBV-positive large B-cell lymphoma emerges after T-cell lymphoma,^40^ occurring sometimes as a clinical relapse without detectable T-cell lymphoma^8^ or being identified simultaneously with T-cell lymphoma.^40^ It has been proposed that latent EBV infection in B cells might be due to immunodeficiency caused by preceding T-cell lymphoma,^13^^,^^15^^,^^40^^,^^41^ or the chronic EBV infection in B cells might have occurred earlier, resulting in viral antigen-driven T-cell proliferation before evolving into a T-cell clone.^12^^,^^13^ However, this EBV mechanism cannot fully explain the development of composite FL and nTFHL, although 2 of our cases were associated with latent EBV infection. In composite FL and nTFHL, both B-cell and T-cell components originate from the follicle center, a special niche with a spatial architecture framed by follicular dendritic cells. Here, follicular B cells and T-follicular helper cells are exposed to the same pathogenic microenvironment, which may provide a common ground for parallel clones to evolve.^42^^,^^43^ It is conceivable that FL precedes nTFHL and immunogenically stimulates prolonged activation of T-follicular helper cells, thereby increasing the risk of mutagenesis in genes such as *TET2*, *DNMT3A*, and *RHOA*, ultimately driving T-cell lymphomagenesis. This hypothesis may explain the zonal distribution observed in this series and a previous case report.^29^ B-cell clones dominate the follicles, while T-cell clones are confined to the T-zone. This contrasts with the diffusely intermixed patterns typically seen in EBV-driven DLBCL-AITL CL.^13^^,^^40^^,^^41^ To date, B-cell and T-cell components in CL are believed to be clonally unrelated, although rare examples of transdifferentiation or lineage switch have been documented in B-cell lymphoma.^44‐46^ In case 1 of our series, *IGH::BCL2* fusion was harbored by FL but not by nTFHL, supporting clonal independence. In cases 2 and 3, NGS studies identified mutation profiles consistent with nTFHL. Notably, in case 3, VAFs of *TET2* and *DNMT3A* mutations were higher than the tumor burden of either T-cell or B-cell components in isolation, suggesting early mutations shared by the 2 neoplasms.^43^  *RHOA* alterations are considered late events in the development of nTFHL. *RHOA* encodes a GTPase, and its mutation enhances signal transduction in CD4 subset T cells, driving their differentiation along the follicular helper cell pathway and promoting their neoplastic transformation.^47^ In contrast, *TET2* and *DNMT3A* belong to epigenetic modifiers, and both mutations occur early in hematopoietic progenitors, leading to dysregulated DNA methylation and perturbed gene expression in hematopoietic stem cells. These early mutations are often shared among parallel subclones in leukemia^48^ or lymphoma^34^^,^^43^ according to recent genomic analyses. However, in the latter case of our series, we cannot exclude the possibility of a copy number alteration, such as simultaneous loss of chromosome 4q (*TET2* locus) and chromosome 2p (*DNMT3A* locus). In future studies, genomic analysis may be performed on sorted cell samples or spatial genomics employed in the evaluation of CL to help reconstruct phylogenic trees and elucidate the clonal relationship between the 2 concurrent neoplasms. Alternatively, analyzing chronologically different samples with varied neoplastic components in individual patients (ie, case 2 in this series) may be helpful in determining the clonal relationship.^3^

In CL of B-cell and T-cell origin, clinical outcome is often driven by the more aggressive component, typically the T-cell lymphoma. Treatment usually targets the T-cell component,^15^^,^^41^ although some regimens may address both lineages.^3^^,^^8^^,^^10^ Literature suggests that relapse can occur as a single-lineage lymphoma, depending on which component survives treatment.^3^^,^^8^^,^^10^^,^^13^ In our series, all 3 patients exhibited aggressive clinical courses. Two patients started chemotherapy against T-cell lymphoma, and the other received palliative care. Of the 2 patients who received chemotherapy, 1 (case 2) demonstrated an initial response but relapsed with isolated T-cell lymphoma 1 year later. Optimal treatment strategies for CL of B-cell and T-cell origins remain to be defined.

## CONCLUSION

Composite lymphoma comprising FL and nTFHL is extremely rare and diagnostically challenging. These lymphomas tend to exhibit zonal distribution, with FL residing in follicles (B-zone) and nTFHL contained in interfollicular areas (T-zone). While the diagnosis of FL is usually straightforward, identifying the neoplastic T-cell component, especially in the absence of flow cytometry, is more difficult. The essential diagnostic features of nTFHL include (1) clusters of abnormal T cells with the TFH phenotype on immunohistochemistry, (2) flow cytometric evidence of aberrant T cells, and (3) an NGS mutation profile associated with nTFHL, which may serve as an alternative to flow cytometry when the latter is unavailable. Clonal TCR rearrangement, latent EBV infection, and high proliferation index in the T-zone are supportive findings for the diagnosis but should be interpreted with caution and in the context of histologic and phenotypic findings. The pathogenesis of composite FL and nTFHL remains uncertain and warrants further investigation.

## Supplementary Material

aqag012_Supplementary_Data

## Data Availability

Data sharing is not applicable as no new data were generated.

## References

[1] Kim H , Hendrickson R, Dorfman RF. Composite lymphoma. Cancer. 1977;40:959-976. 10.1002/1097-0142(197709)40:3<959::aid-cncr2820400302>3.0.co; 2-3 332325 10.1002/1097-0142(197709)40:3<959::aid-cncr2820400302>3.0.co;2-3

[2] Whitling NA , Shanesmith RP, Jacob L, et al. Composite lymphoma of mycosis fungoides and cutaneous small B-cell lymphoma in a 73-year-old male patient. Hum Pathol. 2013;44:670-675. 10.1016/j.humpath.2012.09.014 23313307

[3] Trimech M , Letourneau A, Missiaglia E, et al. Angioimmunoblastic T-cell lymphoma and chronic lymphocytic leukemia/small lymphocytic lymphoma: a novel form of composite lymphoma potentially mimicking Richter syndrome. Am J Surg Pathol. 2021;45:773-786. 10.1097/PAS.0000000000001646 33739791

[4] Tanaka J , Su P, Luedke C, et al. Composite lymphoma of follicular B-cell and peripheral T-cell types with distinct zone distribution in a 75-year-old male patient: a case study. Hum Pathol. 2018;76:110-116. 10.1016/j.humpath.2017.11.017 29217426

[5] Tabata R , Tabata C, Yasumizu R, Kojima M. Independent growth of diffuse large B cell lymphoma and angioimmunoblastic T cell lymphoma originating from composite lymphoma. Ann Hematol. 2014;93:1801-1803. 10.1007/s00277-014-2018-z 24488225

[6] Papalas JA , Puri PK, Sebastian S, Wang E. Primary cutaneous, composite, Epstein-Barr virus-associated, diffuse large B-cell lymphoma and peripheral T-cell lymphoma. Am J Dermatopathol. 2011;33:719-725. 10.1097/DAD.0b013e3181fe363b 21946762

[7] Nagai S , Hiraga J, Suzuki N, et al. Composite lymphoma comprising extranodal NK/T-cell lymphoma and diffuse large B-cell lymphoma. Case Rep Hematol . 2018;2018:1583925. 10.1155/2018/1583925 30515337 PMC6234446

[8] Kawai H , Matsushita H, Kawakami S, et al. A case of composite lymphoma with extranodal NK/T-cell lymphoma, nasal-type and diffuse large B-cell lymphoma. J Clin Exp Hematop. 2019;59:34-39. 10.3960/jslrt.18038 30918142 PMC6528137

[9] Hirose Y , Fukushima T, Masaki Y, et al. Epstein-Barr virus-associated composite lymphoma composed of peripheral T-cell lymphoma and an anaplastic variant of a diffuse large B-cell type of non-Hodgkin’s lymphoma and strongly expressing p53 protein. Int J Hematol. 2004;79:260-265. 10.1532/ijh97.03156 15168595

[10] Du Z , Chen J, Zhou X, Zhang T, Chen B, Tang F. Composite lymphoma with relapse of enteropathy-type T-cell lymphoma. Leuk Lymphoma. 2009;50:749-756. 10.1080/10428190902795519 19330653

[11] Cui W , Fan F, Zhang D, Garnett D, Tilzer L. Primary composite lymphoma of the larynx, composed of diffuse large B-cell lymphoma and peripheral T-cell lymphoma, not otherwise specified, presenting as left subglottic tracheal fistula, esophageal diverticulum, and neck abscess. Ann Clin Lab Sci. 2012;42:73-80. 22371913

[12] Chen YK , Huang E, Lin CC, et al. Composite lymphoma: angiocentric T-cell lymphoma (CD8+ cytotoxic/suppressor T-cell) and diffuse large B-cell lymphoma associated with EBV, and presenting clinically as a midfacial necrotizing lesion. Oral Oncol. 2004;40:353-359. 10.1016/j.oraloncology.2003.09.001 14747069

[13] Wang E , Papavassiliou P, Wang AR, et al. Composite lymphoid neoplasm of B-cell and T-cell origins: a pathologic study of 14 cases. Hum Pathol. 2014;45:768-784. 10.1016/j.humpath.2013.11.008 24565206

[14] Demurtas A , Aliberti S, Bonello L, et al. Usefulness of multiparametric flow cytometry in detecting composite lymphoma: study of 17 cases in a 12-year period. Am J Clin Pathol. 2011;135:541-555. 10.1309/AJCPQKE25ADCFZWN 21411776

[15] Xu Y , McKenna RW, Hoang MP, Collins RH, Kroft SH. Composite angioimmunoblastic T-cell lymphoma and diffuse large B-cell lymphoma: a case report and review of the literature. Am J Clin Pathol. 2002;118:848-854. 10.1309/VD2D-98ME-MB3F-WH34 12472277

[16] Yamazaki S , Fujioka Y, Nakamura F, et al. Composite diffuse large B-cell lymphoma and CD20-positive peripheral T-cell lymphoma. Pathol Int. 2011;61:662-666. 10.1111/j.1440-1827.2011.02713.x 22029677

[17] Zhang B , Zhang Y, Li Q, et al. Case report: chronic lymphocytic leukemia/small lymphocytic lymphoma and monomorphic epitheliotropic intestinal T-cell lymphoma: a composite lymphoma. Pathol Oncol Res. 2022;28:1610653. 10.3389/pore.2022.1610653 36567979 PMC9768801

[18] Miyagawa F , Nakajima A, Ogawa K, et al. Composite EBV-negative marginal zone lymphoma and angioimmunoblastic T-cell lymphoma presenting as multiple subcutaneous nodules. Eur J Dermatol. 2020;30:427-429. 10.1684/ejd.2020.3810 32618566

[19] Gonzalez-Gascon YMI , Menarguez J, Kwon M, et al. Composite lymphoma containing mantle cell and peripheral T-cell lymphoma, not otherwise specified: a report of 2 cases treated with up-front autologous stem cell transplantation. Appl Immunohistochem Mol Morphol. 2020;28:e94-e98. 10.1097/PAI.0000000000000769 30973352

[20] Ichikawa A , Miyoshi H, Yamauchi T, et al. Composite lymphoma of peripheral T-cell lymphoma and Hodgkin lymphoma, mixed cellularity type; pathological and molecular analysis. Pathol Int. 2017;67:194-201. 10.1111/pin.12515 28191697

[21] Huang Y , Hu S, Larson DP, et al. Composite classic Hodgkin lymphoma and follicular lymphoma: a clinicopathologic study of 22 cases with review of 27 additional cases in the literature. Am J Surg Pathol. 2022;46:793-800. 10.1097/PAS.0000000000001828 35067515

[22] Wang S , Tzankov A, Xu-Monette ZY, et al. Clonally related composite follicular lymphoma and mantle cell lymphoma with clinicopathologic features and biological implications. Hum Pathol. 2013;44:2658-2667. 10.1016/j.humpath.2013.07.007 24071012

[23] Miyazawa Y , Yokohama A, Ishizaki T, et al. Pathological and molecular analysis of a composite lymphoma of mantle cell lymphoma and Epstein-Barr virus-positive follicular lymphoma. Int J Hematol. 2021;113:592-599. 10.1007/s12185-020-03035-0 33387297

[24] Liu Y , Li P, Guo Y, et al. A unique composite follicular lymphoma and mantle cell lymphoma with a mixed cell pattern and aggressive course. Am J Clin Pathol. 2014;141:737-741. 10.1309/AJCPAWDDYO5OQYCD 24713749

[25] Jelloul FZ , Chen QH, Yang T, et al. Composite small lymphocytic lymphoma/chronic lymphocytic leukemia and follicular lymphoma: a clinicopathological study of six cases. Int J Surg Pathol. 2018;26:135-144. 10.1177/1066896917737161 29069998

[26] Miyaoka M , Kikuchi T, Carreras J, et al. Composite follicular lymphoma and CD5-positive nodal marginal zone lymphoma. J Clin Exp Hematop. 2016;56:55-58. 10.3960/jslrt.56.55 27334859 PMC6144277

[27] Ryder CB , Saeed H, Hussaini M. Composite lymphoma with follicular lymphoma transformation to clonally related Epstein-Barr virus (EBV) positive diffuse large B-cell lymphoma and EBV-positive classic Hodgkin lymphoma. Case Rep Hematol. 2023;2023:8833273. 10.1155/2023/8833273 38028985 PMC10651334

[28] O’Neill JP , Quinn F, Dowling A, et al. Composite t(14; 18)-negative follicular lymphoma and nodular lymphocyte-predominant Hodgkin lymphoma. Case Rep Hematol. 2018;2018:4312594. 10.1155/2018/4312594 30155322 PMC6098847

[29] Yang Q , Zhang T, Fang N, Sun W. Composite ALK-negative anaplastic large cell lymphoma and follicular lymphoma involving jejunum and mesenteric lymph nodes. Pathol Int. 2024;74:356-358. 10.1111/pin.13433 38656535

[30] Raychaudhuri S , Dong ZM, Knowles S, Graf S. EBV-positive classic Hodgkin lymphoma and primary nodal T-cell/NK-cell lymphoma arising in the background of follicular lymphoma. Case Rep Hematol. 2024;2024:8810646. 10.1155/2024/8810646 39290203 PMC11407883

[31] Alaggio R , Amador C, Anagnostopoulos I, et al. The 5th edition of the World Health Organization Classification of Haematolymphoid Tumours: lymphoid neoplasms. Leukemia. 2022;36:1720-1748. 10.1038/s41375-022-01620-2 35732829 PMC9214472

[32] Campo E , Jaffe ES, Cook JR, et al. The International Consensus Classification of mature lymphoid neoplasms: a report from the clinical advisory committee. Blood. 2022;140:1229-1253. 10.1182/blood.2022015851 35653592 PMC9479027

[33] Kim H. Composite lymphoma and related disorders. Am J Clin Pathol. 1993;99:445-451. 10.1093/ajcp/99.4.445 8475911

[34] Yao WQ , Wu F, Zhang W, et al. Angioimmunoblastic T-cell lymphoma contains multiple clonal T-cell populations derived from a common TET2 mutant progenitor cell. J Pathol. 2020;250:346-357. 10.1002/path.5376 31859368 PMC7064999

[35] Palomero T , Couronne L, Khiabanian H, et al. Recurrent mutations in epigenetic regulators, RHOA and FYN kinase in peripheral T cell lymphomas. Nat Genet. 2014;46:166-170. 10.1038/ng.2873 24413734 PMC3963408

[36] Mozas P , Lopez C, Grau M, et al. Genomic landscape of follicular lymphoma across a wide spectrum of clinical behaviors. Hematol Oncol. 2023;41:631-643. 10.1002/hon.3132 36994552

[37] Guan T , Zhang M, Liu X, et al. Circulating tumor DNA mutation profile is associated with the prognosis and treatment response of Chinese patients with newly diagnosed diffuse large B-cell lymphoma. Front Oncol. 2022;12:1003957. 10.3389/fonc.2022.1003957 36465410 PMC9713409

[38] Delfau-Larue MH , Laroche L, Wechsler J, et al. Diagnostic value of dominant T-cell clones in peripheral blood in 363 patients presenting consecutively with a clinical suspicion of cutaneous lymphoma. Blood. 2000;96:2987-2992. 11049975

[39] Kim TY , Min GJ, Jeon YW, et al. Impact of Epstein-Barr virus on peripheral T-cell lymphoma not otherwise specified and angioimmunoblastic T-cell lymphoma. Front Oncol. 2021;11:797028. 10.3389/fonc.2021.797028 35087758 PMC8786732

[40] Zettl A , Lee SS, Rudiger T, et al. Epstein-Barr virus-associated B-cell lymphoproliferative disorders in angloimmunoblastic T-cell lymphoma and peripheral T-cell lymphoma, unspecified. Am J Clin Pathol. 2002;117:368-379. 10.1309/6UTX-GVC0-12ND-JJEU 11888076

[41] Suefuji N , Niino D, Arakawa F, et al. Clinicopathological analysis of a composite lymphoma containing both T- and B-cell lymphomas. Pathol Int. 2012;62:690-698. 10.1111/j.1440-1827.2012.02858.x 23005596

[42] Kumar E , Pickard L, Okosun J. Pathogenesis of follicular lymphoma: genetics to the microenvironment to clinical translation. Br J Haematol. 2021;194:810-821. 10.1111/bjh.17383 33694181

[43] Attygalle AD , Dobson R, Chak PK, et al. Parallel evolution of two distinct lymphoid proliferations in clonal haematopoiesis. Histopathology. 2022;80:847-858. 10.1111/his.14619 35064935 PMC9310594

[44] Wang E , Papalas J, Hutchinson CB, et al. Sequential development of histiocytic sarcoma and diffuse large B-cell lymphoma in a patient with a remote history of follicular lymphoma with genotypic evidence of a clonal relationship: a divergent (bilineal) neoplastic transformation of an indolent B-cell lymphoma in a single individual. Am J Surg Pathol. 2011;35:457-463. 10.1097/PAS.0b013e3182098799 21317718

[45] Fraser CR , Wang W, Gomez M, et al. Transformation of chronic lymphocytic leukemia/small lymphocytic lymphoma to interdigitating dendritic cell sarcoma: evidence for transdifferentiation of the lymphoma clone. Am J Clin Pathol. 2009;132:928-939. 10.1309/AJCPWQ0I0DGXBMHO 19926586

[46] Feldman AL , Arber DA, Pittaluga S, et al. Clonally related follicular lymphomas and histiocytic/dendritic cell sarcomas: evidence for transdifferentiation of the follicular lymphoma clone. Blood. 2008;111:5433-5439. 10.1182/blood-2007-11-124792 18272816 PMC2424145

[47] Fujisawa M , Sakata-Yanagimoto M, Nishizawa S, et al. Activation of RHOA-VAV1 signaling in angioimmunoblastic T-cell lymphoma. Leukemia. 2018;32:694-702. 10.1038/leu.2017.273 28832024 PMC5843900

[48] Grimwade D , Ivey A, Huntly BJ. Molecular landscape of acute myeloid leukemia in younger adults and its clinical relevance. Blood. 2016;127:29-41. 10.1182/blood-2015-07-604496 26660431 PMC4705608

