# Supplementary material for: Composite lymphoma composed of follicular lymphoma and nodal T-follicular helper cell lymphoma: report of 3 cases highlighting histopathologic zonation of each neoplastic component
Source: Am J Clin Pathol. 2026 Mar 5;165(3):aqag012. doi: 10.1093/ajcp/aqag012 (PMC13016991; doi:10.1093/ajcp/aqag012)
Supplement: aqag012_Supplementary_Data [file aqag012_supplementary_data.zip › ajcp-2025-11-0586-File009.doc]

**Supplemental Table 1. Clinicopathologic features of follicular lymphoma with expanded reactive T-follicular helper cells (control cases)**

| Case number | 1 | 2 | 3 | 4 | 5 | 6 |
| --- | --- | --- | --- | --- | --- | --- |
| Age | 49 | 60 | 78 | 81 | 74 | 17 |
| Sex | Male | Female | Female | Female | Female | Female |
| Clinical presentation | B-symptoms: weight loss, night sweat | SLE; neck mass; no B-symptoms | RCC;  no B-symptoms | T/HR-DLBCL;  B-symptoms: weight loss, fatigue | B-symptoms: weight loss, night sweat | no B-symptoms |
| Radiological findings | mesenteric and retroperitoneal adenopathy | Generalized lymphadenopathy | Generalized lymphadenopathy | Generalized lymphadenopathy | Neck mass | Axillary mass |
| CBC(WBC/Hgb/plt) | NA | NA | Unremarkable | 4.07/13.3/120 | 6.97/11.3/195 | NA |
| Lymphocytosis | NA | NA | No | No | No | NA |
| Serum LDH | NA | NA | 38 | 199 | 239 | NA |
| Biopsied nodal site | Axillary | cervical node | retroperitoneal | axillary | cervical node | axillary node |
| Histopathology | Indistinct T-zone | Indistinct T-zone | Indistinct T-zone | Indistinct T-zone | Indistinct T-zone | Indistinct T-zone |
| B-cell distribution | Follicular | Follicular | Follicular | Follicular | Follicular | Follicular |
| Histologic grade | 3A | 3B, focally | 1-2 | 3A | 3A | Pediatric type |
| Proliferation index | 70% | ~90% focally | ~25% | 30% | 30-50% | 80% |
| B-cell phenotype | CD10-, BCL2+/- | CD10+, BCL2+/- | CD10++, BCL2- | CD10-, BCL2- | CD10+, BCL2-/+ | CD10+, BCL2-/+ |
| T-cell distribution | perifollicular | intrafollicular | intrafollicular | intrafollicular | intrafollicular | intrafollicular |
| Cytologic atypia | No | No | No | No | No | No |
| Microenvironment | No | No | No | No | No | No |
| Proliferation index | 15% | 15% | 10% | NA | 15% | 20% |
| *T-cell phenotype* | CD4+, BCL6+, partial CD10+, PD1+; no loss of T antigens | CD4+, CD10+, PD1+, ICOS+; no loss of pan-T antigens | CD4+, CD10+, PD1+; no loss of pan-T antigens | CD4+, CD10+, PD1+; no loss of pan-T antigens | CD4+, CD10+, PD1+, ICOS+; no loss of pan-T antigens | CD4+, CD10+, PD1+, ICOS+; no loss of pan-T antigens |
| EBV-EBER CISH | Negative | Negative | ND | Negative | Negative | Negative |
| Flow cytometry | Both components detected | Both components detected | ND |  | ND |  |
| B-cells | 28% monoclonal B-cells, CD10- | 25% monoclonal B-cells, CD10+ | ND | 11% monoclonal B-cells, CD10- | ND | 3% monoclonal B-cells, CD10+ |
| T-cells | Negative | 2% CD10-positive T-cells without loss of pan-T antigens | ND | Negative | ND | 3% CD10+ T cells without loss of pan-T antigens |
| B-cell clonality | Clonal *IGH, IGK* | ND | ND | ND | *IGH-, IGK-* | Clonal *IGH* (*IGK*-) |
| T-cell clonality | *TCRG-, TCRB-* | Clonal *TCRG* (*TCRB-*) | Clonal *TCRG, TCRB* | *TCRG-, TCRB-* | *TCRG*-, *TCRB*- | ND |
| FISH analysis | Negative for *IGH::BCL2*, *BCL6*^R^ & *MYC*^R^ | Negative for *IGH::BCL2*, *BCL6*^R^ & *DUSP22::IRF4* | Negative for *IGH::BCL2*, *BCL6*^R^ & *MYC*^R^ | Positive for *BCL6*^R^, negative for *BCL2*^R^ & *IRF4*^R^ | Negative for *IGH::BCL2*, *BCL6*^R^ & *MYC*^R^ | Negative for *IGH::BCL2*, *BCL6*^R^ & *MYC*^R^ |
| NGS analysis | ND | *EBF1* (30.6%); *PIK3CA* (19.6%); *TNFAIP3* (39.4%); *TNFRSF14* (41.7%) | ND | *ARID1A* (9.4%); *KMT2D* x2 (17.2%, 15%); *NOTCH2* (6.9%); *TET2* x2 (16.6%, 16.4%); *TNFAIP3* (35.5%) | No pathogenic mutations detected | *CXCR4* (7.5%); *MAP2K1* (24.3%); *TNFRSF14* (10.7%); *EP300* (26.5%); *KMT2D* (8.6%); *STAT6* (6.1%) |
| Bone marrow biopsy | No lymphomatous involvement | ND | ND | No lymphomatous involvement | ND | ND |
| Treatment | R-CHOP, Bendamustine-R, CAR-T | clinical trial | Bendamustine-R | tafasitamab, revlimid, then ioncastuximab | Lenalidomide, obinutuzumab | NA |
| Follow up (months) | 48 | 21 | 18 | 17 | 3 | 3 |
| Outcome | Relapse; loss of FU | CR | CR | CR | Alive | Loss of FU |

CBC, complete blood cell count; WBC, white blood cells (= × 10^9^/L); Hgb, hemoglobin (= g/L); Plt, Platelets (=× 10^9^/L); LDH, lactase dehydrogenase; FISH, fluorescence in situ hybridization; NGS, Next Generation Sequencing analysis; TRBC1, T-cell receptor beta chain constant region 1; *IGH*, immunoglobulin heavy chain gene; *IGK*, immunoglobulin kappa light chain gene; *TCRG*, T-cell receptor gamma gene; *TCRB*, T-cell receptor beta gene; SLE, systemic lupus erythematosus; RCC, renal cell carcinoma; T/HR-DLBCL, T-cell/histiocyte rich large B-cell lymphoma; R, Rituximab; CHOP, Cyclophosphamide, Doxorubicin (Adriamycin), Vincristine (Oncovin) and Prednisone; CAR-T, chimeric antigen receptor T-cell therapy; NA, not available; ND, not done; +, positive; -, negative; *X*^R^, gene rearrangement; CR, Complete remission; FU, follow up
