# Supplementary material for: Composite lymphoma composed of follicular lymphoma and nodal T-follicular helper cell lymphoma: report of 3 cases highlighting histopathologic zonation of each neoplastic component
Source: Am J Clin Pathol. 2026 Mar 5;165(3):aqag012. doi: 10.1093/ajcp/aqag012 (PMC13016991; doi:10.1093/ajcp/aqag012)
Supplement: aqag012_Supplementary_Data [file aqag012_supplementary_data.zip › ajcp-2025-11-0586-File010.doc]

**Supplemental Table 2. Comparison of clinicopathologic features between follicular lymphoma composite with nodal T-follicular helper cell lymphoma (studied cases) and follicular lymphoma with expended reactive T-follicular helper cells (control cases)**

|  | Cases | Control | *P* value |
| --- | --- | --- | --- |
| Number of cases | 3 | 6 |  |
| Age; median (range) | 75 (49-84) | 67 (17-81) | 0.5169^2^ |
| Sex (M/F) | 2/1 | 1/5 | 0.2262^1^ |
| B-symptoms | 3 (100%) | 3 (50%) | 0.4643^1^ |
| Generalized adenopathy (PET/CT) | 3 (100%) | 4 (66.7%) | 0.5000^1^ |
| Lymphocytosis | 0/1 (0%) | 0/4 (0%) |  |
| Serum LDH (U/L) | 223 | 199 (38-239) |  |
| Histopathology (distinct zonation) | 3 (100%) | 0 (0%) | 0.0119^1^ |
| *B-zone* |  |  |  |
| Follicular B-cell distribution | 3 (100%) | 6 (100%) |  |
| Low grade histology | 2 (66.7%) | 1 (16.7%) | 0.2262^1^ |
| Proliferation index > 20% | 2 (66.7%) | 6 (100%) | 0.3333^1^ |
| Strong BCL2 in follicular B-cells | 1 (33.3%) | 0 (0%) | 0.0833^1^ |
| *T-zone* |  |  |  |
| Peri- or interfollicular T-cells | 3 (100%) | 1 (17.7%)* | 0.0476^1^ |
| Intrafollicular T-cells | 0 (0%) | 5 (83.3%) | 0.0476^1^ |
| Cytologic atypia of T-cells | 3 (100%) | 0 (0%) | 0.0119 |
| Microenvironment in T-zone*** | 3 (100%) | 0 (0%) | 0.0119 |
| Proliferation index in T-zone | 35% (30-65%) | 12.5% (10-20%) | 0.0219^2^ |
| Aberrant T-cell phenotype | 3 (100%) | 0 (0%) | 0.0119^1^ |
| EBV-EBER CISH | 2 (66.7%) | 0/5 (0%) | 0.1071^1^ |
| Biclonal detection by flow cytometry | 3 (100%) | 0/4 (0%) | 0.0286^1^ |
| Clonal *IG* rearrangement | 3 (100%) | 2/3 (66.7%) | 1.0000^1^ |
| Clonal *TCR* rearrangement | 3 (100%) | 2/5 (40%) | 0.1964^1^ |
| *IGH::BCL2* by FISH | 1 (33.3%) | 0 (0%) |  |
| *BCL6* rearrangement | 0 (0%) | 1 (16.7%) |  |
| Mutation profile of Tfh cell lymphoma | 2/2 (100%) | 0/4 (0%)** | 0.0667^1^ |
| Marrow involvement by T-cell lymphoma | 0 (0%) | 0/2 (0%) |  |
| Follow up (months) | 6 (3-24) | 17.5 (3-48) |  |
| Progression or relapse after treatment | 3 (100%) | 1/5 (16.7%) | 0.1429^1^ |

M, male; F, female; PET/CT, positron emission tomography/computed tomography; LDH, lactase dehydrogenase; FISH, fluorescence in situ hybridization; *IG*, immunoglobulin gene; *TCR*, T-cell receptor gene; Ffh, T-Follicular Helper

*One of the two display mixed intrafollicular and interfollicular distribution of T-follicular helper cells, with the former pattern predominant

**Three out of four cases demonstrate mutation profile consistent with follicular lymphoma, while the remaining one shows no pathogenic mutations

***Microenvironment resembling angioimmunoblastic lymphoma: eosinophilic infiltration, increased endothelial venules, expanded follicular dendritic meshwork, etc.
